# Intra-Individual Behavioural Variability: A Trait under Genetic Control

**DOI:** 10.3390/ijms21218069

**Published:** 2020-10-29

**Authors:** Rie Henriksen, Andrey Höglund, Jesper Fogelholm, Robin Abbey-Lee, Martin Johnsson, Niels J. Dingemanse, Dominic Wright

**Affiliations:** 1 AVIAN Behavioural Genomics and Physiology Group, IFM Biology, Linköping University, 58183 Linköping, Sweden; andrey.hoglund@liu.se (A.H.); robin.abbey-lee@liu.se (R.A.-L.); martin.johnsson@slu.se (M.J.); 2 The Roslin Institute and Royal (Dick) School of Veterinary Studies, The University of Edinburgh, Edinburgh EH25 9RG, UK; 3 Department of Animal Breeding and Genetics, Swedish University of Agricultural Sciences, 750 07 Uppsala, Sweden; 4 Ludwig Maximilians University of Munich (LMU), 82152 Munich, Planegg-Martinsried, Germany; n.dingemanse@lmu.de

**Keywords:** individual differences, QTL, Gallus gallus, fearfulness, sociability, personality

## Abstract

When individuals are measured more than once in the same context they do not behave in exactly the same way each time. The degree of predictability differs between individuals, with some individuals showing low levels of variation around their behavioural mean while others show high levels of variation. This intra-individual variability in behaviour has received much less attention than between-individual variability in behaviour, and very little is known about the underlying mechanisms that affect this potentially large but understudied component of behavioural variation. In this study, we combine standardized behavioural tests in a chicken intercross to estimate intra-individual behavioural variability with a large-scale genomics analysis to identify genes affecting intra-individual behavioural variability in an avian population. We used a variety of different anxiety-related behavioural phenotypes for this purpose. Our study shows that intra-individual variability in behaviour has a direct genetic basis that is largely unique compared to the genetic architecture for the standard behavioural measures they are based on (at least in the detected quantitative trait locus). We identify six suggestive candidate genes that may underpin differences in intra-individual behavioural variability, with several of these candidates having previously been linked to behaviour and mental health. These findings demonstrate that intra-individual variability in behaviour appears to be a heritable trait in and of itself on which evolution can act.

## 1. Introduction

Individuals within a population are often repeatable in many aspects of their behaviour [1]. Repeatable differences in behavioural traits, such as aggressiveness, shyness, sociability and activity between individuals have given rise to a growing field focusing on animal personality and have motivated the development of many evolutionary theories aimed at understanding the processes that allow and maintain such within-population variation [2].

Individual repeatability does not mean that the behaviour of an individual is fully predictable or stable. An increasing number of studies have shown that when individuals are measured more than once in the same context they do not behave in exactly the same way each time [3]. Interestingly, the degree of intra-individual variation also differs between individuals, with some individuals showing low levels of variation around their behavioural mean while others show high levels of variation [4]. This can result both from intrinsic individual variability in behavioural or individual differences in phenotypic plasticity in response to unmeasured internal or external stimuli [5]. Whereas proximate and ultimate causes of inter-individual variation in behaviour has been an area of intense research interest across animal taxa [6], intra-individual variation in behaviour has previously been assumed to be homogenous across individuals. Interest in this field has increased, however. A variety of studies have assessed the extent of this variation in species and how it can affect ecologically relevant situations [7,8,9,10,11,12,13]. Despite this interest, almost nothing is known about the genetic factors that affect this large but understudied component of behavioural variation [14,15,16], with only one study assessing heritability in this to date [17]. Insight into the potential genetic control that gives rise to intra-individual behavioural variability is crucial for disentangling its proximate causes and thereby fine-tune tests of hypothesis about the evolution of this type of behavioural variability [18]. Knowledge of the genetic basis of intra-individual behavioural variability can also help understand the link between personality and behavioural variability, and to what extent and how intra-individual behavioural variability is consistent in different situations.

A classical approach to identify the genetic regions or loci that underpin a trait is to use a technique known as quantitative trait locus (QTL) mapping. This is a process by which two populations divergent for the trait of interest are inter-crossed for two or more generations, to create an F_2_ or further intercross. This intercross can then be used to map genetic loci affecting the trait of interest by genotyping the individuals using markers that can differentiate between the two parental populations. These markers can then be used to map the larger-effect loci that underpin the variation between the two parental populations. This approach can be further complemented by using an advanced intercross (intercrossing for additional generations to generate more recombinations) and integrating gene expression (expression QTL) to further aid in gene identification. Such intercross populations are poor at calculating the heritability of the founder populations (essentially only representing the number of individuals actually used in the initial parental intercross), but offer the ability to identify the actual genomic regions affecting the trait of interest.

In this study, we combine standardized behavioural testing to estimate intra-individual variation in anxiety and sociability related behaviours with a previously generated large-scale combined genetics and genomics analysis to attempt to identify genes affecting intra-individual behavioural variability in a population of intercross chickens. Whereas more typically studies on intra-individual variation restrict themselves to a single trait, here we have replicate measures (two per test) of three different separate anxiogenic tests. This allows us to assess how repeatable intra-individual variation is within and between different contexts and test conditions. By combining quantitative trait locus (QTL) and expression QTL (eQTL) analyses of the brains of an advanced intercross based on Red Junglefowl (the wild progenitor of the modern domestic chicken) and domestic White leghorn chickens we identify putative genes underlying phenotypic differences in intra-individual behavioural variability and to what degree these differ between behavioural trait.

## 2. Methods

### 2.1. Overview of Study Performed

The main objective of this study was to test if intra-individual behavioural variability is trait-specific, what is the genetic architecture of this trait and whether it is discrete from the standard trait QTL, and whether candidate genes for these traits could be identified. To perform this, we used an eighth generation advanced intercross between wild and domestic chickens. These birds had already been used in a genetical genomics experiment, with gene expression measured in the hypothalamus of these birds, and with three separate anxiety-related traits measured: Open field (OF), Social reinstatement (SR), and Tonic immobility (TI). These traits have already been QTL mapped for their mean trait effects [19,20,21], while expression QTL (eQTL) were also measured in these birds, based on the gene expression profiles. eQTL are similar to standard QTL, but the phenotypes used are gene expression values. To identify candidate genes for these anxiety behaviours, first the trait behavioural QTL were mapped, then the eQTL were mapped, before the two sets of QTL and eQTL were overlapped with one another. Any of the eQTL that overlapped with a QTL were considered to be potential candidates. As each individual in the study (*n* = 129) with a gene expression profile was also assayed for anxiety behaviour, it was possible to correlate the expression of each of these overlapping genes with the actual behavioural trait it overlapped with. Any genes that were significantly correlated were then taken forward to be assessed using a statistical causality analysis. This analysis is used to determine whether a genomic location controls gene expression, and that gene expression in turn controls a phenotypic (behavioural) trait. In this way, it is possible to identify genes that putatively control a particular phenotypic trait.

The same approach was therefore applied to the intra-individual variability (IIV) traits that were derived from the anxiety behaviours that were previously mapped. As eQTL and gene expression profiles were also available, it was possible to not only QTL map the IIV traits, but also to overlap them with the eQTL, and test these further to assess for potential candidate genes that regulated anxiety-related IIV behaviours. As the genetic architecture (QTL locations and effects) were already available for the standard trait effects (individual trials and means of the two trials), it is therefore possible to compare the two types of genetic architecture to assess how closely they overlap and how much they differ.

### 2.2. Chicken Study Population and Cross Design

The population used in this study was an eighth generation intercross between a population of Red Junglefowl, derived originally from Thailand and a line of selected White Leghorn chickens [22,23], with a total of 572 F8 individuals used in this study. The intercross was based on 3 White Leghorn (WL) females and 1 Red Junglefowl (RJF) male. These 4 birds were used to generate 41 F1 and then 811 F2 progenies. The 572 F_8_ individuals were produced over six batches and were generated from 118 families using 122 F_7_ individuals (63 females and 59 males), while the average family size was 4.76 ± 3.1 (mean, s.d.) in the F_8_. This advanced intercross has already been used to identify candidate genes underlying variation in anxiety and sociability related behaviours [19,20,21]. The birds were behaviourally tested between the age of 3 and 5 weeks of age (for the social reinstatement and open field assays) and adulthood (for the tonic immobility assay). All individuals were culled by cervical neck dislocation followed by decapitation (as per the ethical permit) at 212 days of age after which the hypothalamus was dissected out and RNA extracted. For further details on feed and housing see [24]. The study was approved by the local Ethical Committee of the Swedish National Board for Laboratory Animals, ethical permit Dnr 50–13.

### 2.3. Behavioural Phenotyping

The advanced intercross birds had previously been phenotyped and mapped for three different behavioural traits: open field behaviour, social reinstatement and tonic immobility, with each measuring some aspects of anxiety-related behaviour. The specific tests are described in detail below:

#### 2.3.1. Social Reinstatement

The social reinstatement test [25] measures social motivation and anxiety, with stressed chicks exhibiting a stronger social cohesion response [26]. In this test, the individual is placed at one end of a narrow arena, with conspecifics located at the far end. The amount of time the individual spends associated with the conspecifics as opposed to exploring the remainder of the arena is considered a measure of sociality and anxiety. A more social or anxious animal will spend more time associating with conspecifics and will approach the conspecifics more rapidly, and therefore spend less time in the start zone of the arena [26]. Trials were performed in a 100 cm × 40 cm arena. The social zone measured 20 cm × 40 cm and was adjacent to a wire mesh compartment containing three unfamiliar conspecific birds of the same age. Birds were placed in the start zone of the arena (also measured 20 cm × 40 cm) in the dark, prior to the lights being turned on and the trial beginning. Measurements were taken using the Ethovision software and continuous video recording (Noldus Information Technology, www.noldus.com). For each trial, total distance moved, length of time spent in the stimulus zone, latency to first enter the stimulus zone, and length of time in the start zone were measured. Each trial was five minutes and replicated twice per individual, with one week between an individual’s first and second test. Individuals were immediately removed from the arena upon the completion of the test to reduce potential habituation. Trials were first performed at 3 weeks of age. There was 1 week between an individual’s first and second trial.

#### 2.3.2. Open Field

The open field assessment is a standard anxiety measurement, where the individual is placed alone in a brightly lit novel area after which behaviour is measured for a fixed duration and has been performed in a variety of vertebrates and invertebrates [27]. In our study, trials were performed in a 100 × 80 cm arena. Individuals were placed in the corner of the arena in complete darkness, prior to the test starting, with the lights turned on immediately at the commencement of the test. Trials lasted 5 min. Measurements were taken using the Ethovision software and continuous video recording (Noldus Information Technology, www.noldus.com). For each trial, total distance moved, proportion of time spent in the central zone (the 60 × 40 cm area in the middle of each arena was considered to be the central zone), velocity, and frequency (number of times) that the central zone was entered were measured. Velocity consists of the average time taken to move between two consecutive time-points, therefore this can distinguish if an animal moves rapidly or slowly through the arena. Each trial was performed twice, with ~1 week between an individual’s first and second trial, with the first trial at 4 weeks of age. The inter-test interval was the same as for the social reinstatement test and the tonic immobility test. Individuals were removed from the arena immediately upon the test finishing to reduce potential habituation.

#### 2.3.3. Tonic Immobility

The third test, the tonic immobility test, measures an individual duration of immobility after being placed on its back and is thought to be a defence strategy evolved to reduce a predator’s interest in the prey, when the prey stops moving after it has been caught. The longer the animal stays in this immobile state the more fearful it is considered to be. In our study, the test bird was placed on its back in a V-shaped wooden cradle (approximately 50 cm in length) and held by the experimenter with one hand over the sternum. The bird was held for 10 s and then the hand was slowly removed. The duration of tonic immobility was recorded up to 600 s. If the bird stood up within 30 s after the hand was removed from its sternum, new attempts to induce tonic immobility were made with up to three attempts per bird (for more information see [19]). The birds were tested after sexual maturity at ~170 days of age, with 7 days between each trial.

### 2.4. Defining Intra-Individual Variability as a Trait

**Figure 1:**
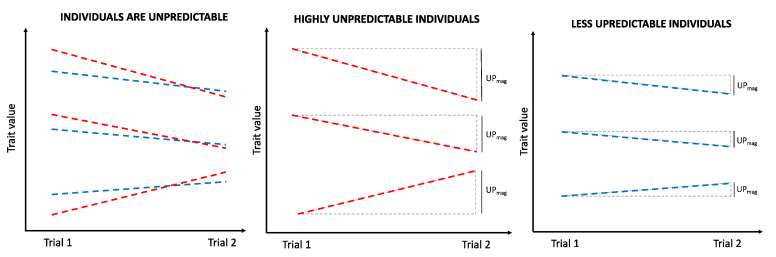
Illustration of intra-individual variation. The magnitude of intra-individual variation was calculated as the absolute difference between the behavioural trait value obtained during the first trial and second trial.

The main objective of this study was to test if intra-individual behavioural variability is a discrete trait, separate to the behaviour itself, and in particular if we can detect genomic loci that underlie this trait. We therefore calculated the degree of intra-individual variability present in the three separate behavioural traits already measured. As each test was performed twice for each bird, it was possible to calculate the magnitudes of the difference between the two trials to give an estimate of the Intra-individual variation (IIV, see Figure 1, and below) for each of the specific sub-phenotypes taken from each test (i.e., the IIV of distance moved in the open field, velocity in the open field, etc.), with increasing magnitude indicating increasing intra-individual variation. Initially the magnitude of intra-individual variation was calculated for each behaviour (IIV_trait_). Second, these calculations of magnitude were used to calculate an average intra-individual variation magnitude per test (IIV_average_) and finally a global magnitude of intra-individual variation was calculated for each individual based on all the recorded behaviours from the three tests (IIV_global_), see below for more details). These composite variables were calculated as a way to see whether combined overall metrics for these traits could also be used to identify general IIV QTL for all sub-traits in a test and even if QTL for general IIV over multiple tests could be identified. Such loci can also be found when multiple different IIV QTL overlap the same location.

### 2.5. Calculating the Magnitude of Intra-Individual Behavioural Variability

Intra-individual variation (IIV) was calculated for each behavioural trait (IIV_trait_) as the absolute difference in values obtained for that trait in trial 1 versus trial 2. For these individual intra-individual trait values, the QTL analysis was run both with and without using the mean trait score as a covariate (see below). An average intra-individual variation (IIV_average_) was calculated for each test situation as the sum of all IIV_trait_ calculated within that test situation. Finally, a global intra-individual variation (IIV_global_) was calculated for each individual based on all the recorded behaviours from the three tests. Trait means and standard deviations are included in Supplementary Materials Table S1. To test for correlations between IIV traits, a Pearson’s correlation test was used.

IIV = Intra-individual variation

IIV_trait_ = | Trait _Trial-1_ – Trait _Trial-2_ |

IIV_average_ = (Sum of all IIV_trait_ within a test)/(Number of traits measured in test)

IIV_global_ = (Sum of all IIV_trait_ for all test)/(Total number of traits measured)

### 2.6. Genotyping and QTL Mapping

A full set of genotypes and the required marker map for QTL mapping was already generated previously (see [28,29] for full details). This map comprised of a total of 652 SNP, that were used to generate a map length of ~9267.5 cm, with an average marker spacing of ~16 cm. Note that the study presented here is an example of a classical linkage study—it uses linkage between markers to map the number of recombinations that occur between two populations that have been intercrossed, with these recombinations occurring in a fixed series of inter-cross generations [30]. In contrast, a Genome-Wide Association Mapping study uses the linkage disequilibrium that exists in a single natural population (and has built-up historical recombinations over a much longer period of time). The advantage of a linkage study is that the genome is covered using relatively few markers. There is not much advantage gained from increasing the marker density to having less than 10 cm between markers, with the recommended marker density for standard QTL mapping being an average distance of 20–30 cm between markers [31]. In the study presented here, we have an average marker density of ~16 cm, therefore we have followed these recommended guidelines. The increase in total number of recombinations present in the intercross is reflected in the threefold increase in total map length of the intercross in the F_8_ compared to the F_2_ generations (~3000 cm in the F_2_ ~9000 cm in the F_8_ generation). QTL analysis was performed using the R/qtl software package [32] and interval mapping was performed using an additive + dominance model. Batch and sex were always included in the QTL behavioural analysis as fixed effects, while a principle component to account for population structure was included as a covariate. In addition, for all the individual trait IIV values, two analyses were run with and without the trait mean as a covariate. This is important to ascertain whether effects are due to the range being proportional to the mean trait value [7]. A sex interaction effect was added, when significant, to account for a particular QTL varying between the sexes. Digenic epistatic analysis was performed according to Broman and Sen (2009) guidelines [33], and a global model incorporating standard main effects, sex interactions, and epistasis was built. The most significant loci were added to the model first, followed by the less significant loci. eQTL mapping was also performed previously on the cross using R/qtl [21]. A local, potentially cis-acting, eQTL (defined as a QTL that was located close to the target gene affected) was called if a signal was detected in the closest flanking markers to the gene in question, to a minimum of 100 cm around the gene (i.e., 50 cm upstream and downstream of the gene). A distance of 50 cm was used to ensure that at least two markers up and downstream from the gene location were selected to enable interval mapping to be performed. The trans-eQTL scan encompassed the whole genome and used a genome-wide empirical significance threshold. In total, 535 local eQTL and 99 trans-eQTL were identified previously.

### 2.7. Significance Thresholds

Significance thresholds for the behavioural QTL analysis were calculated by permutation tests [34,35]. Permutation is by the far the most standard method for calculating significance thresholds in QTL mapping [30], and involve shuffling the phenotype (trait), while maintaining the genotype structure for each individual. For a permuted dataset, a full QTL mapping scan is then conducted and the highest trait value of all the positions assessed is retained. This is then repeated 1000 times, to give 1000 permuted values showing the highest QTL effect detected by chance in each case. To assess significance in the original data, any QTL detected are then compared to the permuted data, with this being used to generate an experiment wide threshold that controls for the large number of tests performed during QTL mapping (i.e., with a marker map of 9000 cm, essentially 9000 tests are being performed, though this is complicated by the fact that many are of course not independent from one another, hence the power of the permutation approach). The function n.perm in the r/qtl package was used to perform these permutations and generate the significance thresholds [32]. A genome-wide 20% threshold was considered suggestive, with this being more conservative than the standard suggestive threshold [36], while a 5% genome-wide level was significant. The ~5% significant threshold was LOD ~4.4, while the suggestive threshold was ~3.6. Confidence intervals for each QTL were calculated with a 1.8 LOD drop method (i.e., where the LOD score on either side of the peak decreases by 1.8 LOD), with such a threshold giving an accurate 95% confidence interval for an intercross type population [37]. The nearest marker to this 1.8 LOD decrease was then used to give the confidence intervals in megabases. Epistatic interactions were also assessed using a permutation threshold generated using R/qtl, with a 20% suggestive and 5% significant genome-wide threshold once again used. In the case of epistatic loci, the approximate average LOD significance threshold for pairs of loci were as follows (using the guidelines given in Broman and Sen [33] full model ~11, full vs. one ~9, interactive ~7, additive ~7, additive vs. one ~4.

Although permutation testing in this manner accurately controls for the large number of multiple testing performed during QTL mapping in terms of the number of markers being tested, it does not control for the number of independent traits being analysed. In this case, we have analysed 13 different traits taken from three separate behavioural tests. Within each test, however, the traits are strongly correlated with one another (see Table 1). As all correlated tests are not independent, they do not need to corrected for. Of the tests, all open field IIV traits were strongly correlated with one another, while social reinstatement IIV traits were essentially composed of two independent groups. Strong correlations also existed between the traits comprised of averages over different tests (global IIV, social reinstatement and open field behaviour IIV). As these composite traits were significantly correlated with the individual IIV measurements and not independent it was not necessary to apply a multiple testing correction for them. Therefore, we applied a multiple testing correction of 4, representing the three different test types, plus one additional correction as social reinstatement essentially comprised of two groups. To correct the LOD thresholds, the log_10_ of 4 was added to the LOD score (one LOD being equivalent to a tenfold increase in significance). This led to final suggestive and significant thresholds of ~4.2 and ~5.0 LOD, respectively.

### 2.8. Candidate Gene Analysis

To identify putatively causal genes underlying intra-individual behavioural variability, candidate QTL were overlapped with eQTL detected in the same cross (see [21]). For this overlap, any eQTL whose 95% confidence interval overlapped with the 95% confidence interval of a behavioural IIV QTL was then identified as being a putative candidate for further analysis. Once these QTL and eQTL were overlapped, each significant eQTL that overlapped a QTL was correlated with the IIV behavioural trait of the QTL to test for significance. For each eQTL overlapping a behaviour QTL, a linear model was fitted with the behaviour trait as a response variable and the expression trait as predictor, including sex and batch as factors. Weight at 42 days was included for traits where weight was used as a covariate in the QTL analysis, all nonsignificant co-factors were then removed sequentially from the model. A multiple testing correction was included based on the number of overlapping eQTL that were present in each QTL region, though where these probes were correlated with one another the multiple testing correction was reduced accordingly due to such probes being non-independent. Any probes that were suggestive (*p*-value below a nominal 0.05 threshold) or significantly correlated (those with a multiple testing corrected *p*-value below 0.05) were then used for the final causality analysis using NEO (see below). One issue with using this approach with this particular data set is that the behavioural QTL were based on up to 572 individuals, whereas the eQTL/expression phenotypes were available only for 129 individuals. Therefore, the network edge orienting (NEO) method for causality testing was applied only where the behavioural QTL that a gene was potentially causative to was detectable in the smaller data set (*n* = 129). This technique has previously been successfully used with this intercross to detect genes that were potentially causal for both open field and social reinstatement behaviour [20,21].

### 2.9. Network Edge Orientation (NEO) Analysis

Causality analysis was performed using NEO software [38] to test whether the expression of correlational candidates was consistent with the transcript having a causal effect on the behavioural trait. Single-marker analysis was performed with NEO fitting a causal model (marker → expression trait → behaviour) and three other types (reactive, confounded, and collider). The models tested by NEO were (1) CAUSAL: Genotype modifies gene expression which in turn modifies behaviour (genotype → expression trait → behaviour). (2) REACTIVE: Genotype modifies behaviour which in turn modifies the expression trait (genotype → behaviour → expression trait). (3) CONFOUNDED: Genotype modifies both the expression trait and the behaviour separately (expression trait ← genotype → behaviour). (4) COLLIDER (behaviour is the collider): Genotype and the expression trait both independently modify behaviour (genotype → behaviour ← expression trait). (5) COLLIDER: (expression is the collider): Genotype and behaviour both independently modify the expression trait (genotype → expression trait ← behaviour). The leo.nb score quantifies the relative probability of the causal model (the preferred model) to the model with the next best fit (of the four remaining). The NEO software evaluates the fit of the model with a *χ*^2^-test, a higher *p*-value indicating a better fit of the model. The best fitting model is chosen based on the ratio of the *χ*^2^ *p*-value to the *p*-value of the next best model on a logarithmic scale (base 10), called local edge orienting against the next best model (leo.nb) scores. A positive leo.nb score indicates that the causal model fits better than any competing model. Aten et al [38] use a leo.nb score of 0.8 when two traits share the same SNP-anchor locus, and a multiple genetic marker leo.nb.oca of 0.3 as their threshold. More recently, other authors have relaxed this to use a threshold of 0.3 for the leo.nb score [39,40]. They also suggest that users inspect the *p*-value of the causal model to ensure the fit is good (in this case meaning the model *p*-value should be non-significant if the causal model fits the best). In effect this *p*-value is the probability of another model fitting the observed data (i.e., if this is significant, then another model also fits the data, if it is not significant then the causal model is the only one that fits). For each gene, we report the leo.nb score and *p*-value of the causal model. Leo.nb scores of 0.3 or more were considered suggestive, whilst leo.nb scores of 1.0 or more were considered significant. Contrastingly, if the *p*-value of the model was non-significant then only the causal model was significant, whilst if this was significant (*p* < 0.05) then another model also fitted the data.

### 2.10. Data Availability

Microarray data for the chicken hypothalamus tissue are available at E-MTAB-3154 in ArrayExpress.

## 3. Results

### 3.1. Correlational Structure between Intra-Individual Behavioural Variability Traits

All of the IIV sub-traits within the Open Field test were strongly correlated with one another, while these traits were also correlated with the IIV of distance moved in the Social Reinstatement test, as well as with the global, average of all OF traits, and average of the combined OF and SR traits (see Table 1). A similar pattern was seen for the Social Reinstatement IIV sub-traits, however one trait (IIV of distance moved in the SR test) was correlated with Open Field IIV traits rather than the other SR IIV traits. In contrast, the Tonic Immobility IIV trait shows no correlation with any of the sub traits from either Open Field or Social Reinstatement tests. The correlations of the different intra-individual behavioural variability scores therefore appear to show that individuals were repeatable in Intra-Individual Variability (IIV) across the SR-test and OF-tests (Table 1). For some behavioural traits, the magnitude of intra-individual variability was even more strongly positively correlated between behaviours in different test-situations, than behaviours within the same test situation (see Table 1). These correlations were seen between IIV scores obtained from the OF-test and the SR-test (such as magnitude of IIV in “movement” in the SR test and magnitude of IIV in “velocity” or “time-spent-in-centre” in the OF test). The same pattern could be seen in the correlations between average IIV scores obtained from the OF-test and SR-test and the individual IIV variability scores obtained from each trait (see Table 1). 

### 3.2. Genetic Architecture of Intra-Individual Behavioural Variation

In total, 13 IIV traits were analysed from the three tests, with four each from the open field test and social reinstatement test, one from the tonic immobility test and four that were created by averaging within and between tests. We identify18 IIV QTL in 10 discrete loci, so essentially 10 separate QTL regions were identified for these traits (see Table 2). Of the initial 18 QTL, twelve QTL were detected for the magnitude of IIV within the OF-test, two QTL for the magnitude of IIV within SR-test, three QTL for the magnitude of IIV for the TI-test and one QTL for “global” magnitude of IIV (an overall measure of IIV using all the behavioural traits—see methods). The average effect sizes of these QTL was 5.8%, which is in line with the standard effect size of a behavioural QTL (Flint and Mott. 2001) and was slightly higher than the average for the main effect QTL for these traits (i.e., the QTL from the mean trait values), which was 5.0%. In terms of the direction of effects (i.e., if the allele conferring the largest effect was domestic or wild-derived), these were fairly equally distributed, with eight showing a greater additive effect coming from the White Leghorn allele, five showing a greater additive effect from the Red Junglefowl allele, and six with only dominance effects. Overall, the detected genetic architecture for IIV was largely separate from the genetic architecture for the standard corresponding traits (see Table 2 and Supplementary Table S2). Only three QTL, located on chromosome 10 (from 126–130 cm), on chromosome 7 (at 104cM) and on chromosome 24 (located at 77 cm) overlapped any of the previously detected QTL for behavioural scores in the OF and SR (see Table 2, and [20,21]). In the case of the QTL on chromosome 24 (for IIV in the open field test), although there was an overlap with a standard QTL, this QTL was for the social reinstatement test, not for the open field test. This pattern held true even when correcting for the mean value of the behavioural traits (see Supplementary Table S3), thereby ensuring that variation in the mean value did not contribute to variation in the variability measured.

### 3.3. Candidate Genes for Intra-Individual Behavioural Variability

To identify potential candidate genes that are involved in forming different IIV phenotypes, we combined quantitative trait locus (QTL) and expression QTL (eQTL) analyses of the brains from the advanced intercross. Intra-individual behavioural variability QTL were overlapped with eQTL, then the relevant behavioural IIV trait was correlated with the overlapped expression trait to identify those that not only overlapped but also exhibited a significant correlation (as used in [20,21]). This initial analysis led to the identification of 10 putative genes underlying phenotypic differences in behavioural IIV, with these genes then taken to the next step of causality analysis (see Table 3).

As correlations alone are not enough to indicate the direction of the relationship between the candidate genes and behavioural traits, and support causality, we then utilized the network edge orienting (NEO) package [38], to infer causality between genotype, gene expression and behavioural IIV. The NEO package uses structural equation modelling to test the fit of five models each explaining a potential relationship between genotype (based on genetic marker), gene expression and behavioural IIV. The analysis produces a leo.nb score and a model *p*-value (see methods). The leo.nb score is defined as the log10 ratio of the causal model *p*-value to the next best model of the four remaining (all of which suggest a non-causal relationship between the behaviour and candidate gene). Therefore, a leo.nb score of 0.3 indicates that the causal model is the best fit, with twice as high model *p*-value than the next best model. In NEO and structural equation modelling in general, a small model *p*-value (e.g., *p* < 0.05) indicates a poor model fit. Our NEO analysis found six of the candidate genes (*Novel gene/X603599288F1, ITGBL1, SFRP4, X603600179F1/LOC100136711, MAP7, ENPP1*) passed the suggestive threshold of 0.3 (see Table 3). The NEO analysis also showed support for the gene C7H2oRF47 as controlling the magnitude of “global” IIV (this model was done with multiple markers, which has a recommended threshold of 0.3, see [38]), with the model almost reaching the more stringent threshold suggested by Aten and colleagues.

Four of the candidate genes had previously been identified as having some bearing on “behaviour” (*MAP7, SRP4,* *ENPP1, LOC100136711*) or neuronal development (*MAP7*), while one had no previous evidence of functionality *(ITGBL1*) and one was a novel gene (*X603599288F1*).

## 4. Discussion

We find that intra-individual variability (IIV) in behaviour is replicable between separate traits measured in the same behavioural test and to some extent between traits in different behavioural tests, although of similar contexts. These cross-test correlations demonstrate that individuals show consistency in their level of behavioural IIV both within and across test-type. While the repeatability of IIV that we find across traits measured within the same behavioural test could be caused by either correlated measurement error, or because they all respond in the same way to the same (unmeasured) environment gradient, the positive correlations we find between IIV across different types of assays are not autocorrelated. Note that the above conclusions can only be from the correlations that exist between the individual IIV measures. The correlations that exist between the different IIV traits and the composite measurements is not an indication of within-trait consistency, as these composite measures are by definition always correlated with the individual sub-traits they are composed of. In addition, we detected 10 discrete genetic loci (reflected in 18 QTL), with only three of these overlapping any of the QTL detected for the standard behavioural measures. Therefore, the majority of the IIV QTL detected in this cross had at least a partially separate genetic architecture to the standard behavioural QTL from the same tests. An important caveat here is that with any QTL analysis, not all of the QTL affecting the trait will be detected, in particular smaller effect QTL will easily be missed [30], and we will have less power to detect QTL that fall between two markers that are relatively far apart. Therefore, many more QTL for the IIV and standard behavioural traits exist but with effect sizes too small to detect, and these may well overlap. Therefore, we are restricted to concluding that of the detected QTL, very few overlap, but that we cannot say this is consistent for the entire genetic architecture.

IIV (also referred to as the variability sensitivity hypothesis), although previously ignored, has more recently been considered more carefully and is now considered a trait in its own right [41,42]. Mechanistically, IIV consists of two aspects of temporal variability [7]: systematic changes and residual variation. Systematic changes in behaviour over time (such as habituation and acclimatization effects, with the animal adjusting/adapting to the test itself) usually occur over a short time period, while “residual” unpredictable variation shows no pattern [43,44]. Both of these types may not simply be noise, but stable instability over time [9,45,46], with evolutionary and ecological advantages. In birds, IIV in song repertoire has been found to decrease with age [9], while poor nutrition in early life can lead to a reduction in IIV later [10]. Provisioning in great tits can also vary in response to the environment, with IIV increasing in challenging conditions [11], whilst brood size manipulations in pied flycatchers can lead to variability sensitivity in provisioning [12]. Unpredictable behaviour has also been shown to reduce capture risk when an individual is under a threat of predation [8,13]. Hermit crabs, for example, increase both their startle response time and their IIV in response to an increase in the perceived risk of predation [8].

Despite these advantages, for IIV to evolve and be selected upon, it must be heritable [47,48]. To date, only one study has estimated the heritability of IIV [17]. This study found a low, yet significantly non-zero heritability estimate of 0.03 (0.02–0.05 interval) for predictability in docility behaviour in marmots. As yet, however, no previous studies have examined the genetic architecture for this type of trait, which we have performed here. We identified a total of 18 QTL to underlie between-individual variation in IIV, of which only three QTL overlapped any of the previously detected QTL for the behavioural scores they were based on. However, the IIV QTL did not overlap the behavioural QTL from the mean behavioural traits they were based on (see Supplementary Materials Tables S2 and S3). This implies that selection can act separately on an individual’s level of fearfulness and degree of IIV in fearfulness, thereby increasing the scope for diversity in behavioural phenotypes [7]. An individual’s fear response would therefore not necessarily be an indication of how predictable that individual would be in its fear response. However, our finding, that individuals show consistency in predictability across test situations, indicates that predictions can be made about an individual’s predictability from one situation to another, at least to some extent. This is particularly interesting in light of the idea of contextual plasticity [49], which implies that IIV should be test specific.

We identified 6 candidate genes underlying intra-individual variation in behaviour. Some of these genes have previously been shown to be involved in natural behavioural variation and neural development, *SFRP4* and *LOC100136711* (the probeset was previously annotated as *LOC770352*, [21]) have, for example, been linked to anxiety in the chicken [21], demonstrating some overlap in the genetic architecture of intra-individual variation in behaviour based on anxiety-related traits and anxiety itself. Another of the candidate genes *ENPP1* has been linked to learning and memory abilities in mice, while *MAP7* has been shown to play a critical role in the developmental regulation of neural axon branch maturation (*MAP7*, [50]) and is also involved in schizophrenia (*MAP7*, [51,52]). This could indicate a potential link between schizophrenia and intra-individual variation in behaviour and the genes that underlie these traits. Increased intra-individual behavioural variability characterizes the performance of people with schizophrenia and has been used for early diagnosis of this disorder (for review see [53]).

The intra-individual behavioural variability we measure in our population could be due to an innate difference in predictability between individuals, or alternatively could be due to inter-individual differences in adjustment to changes in novelty, such as habituation (decreased responsiveness to a stimulus with repeated presentation) or sensitization (increased responsiveness to a stimulus with repeated presentation, [54]). Domestic ducks, for example, habituate faster than wild Mallard ducks [55] and we find slightly more domesticated alleles associated with intra-individual behavioural variation suggesting that the domesticated genotype produces an individual that is either more unpredictable in its behaviour or habituates faster. Domesticated chickens differ considerably in their behavioural responses from their wild progeny the Red Junglefowl, especially in anxiety related tests, such as the ones used in this study [56] and have also developed a brain that differs both in size and composition from their wild progeny [57]. Alternatively, it could also be that the intra-individual behavioural variation that we measure is due to individual differences in behavioural stabilization [5]. However, due to the nature of our data we can only speculate about the true nature of the intra-individual variability that we measure. The fact that we identify an underlying genetic mechanism implies not only that this is a trait that selection can act upon, but also a trait that can interact with and be affected by different environmental factors and potentially also determine the sensitivity of an individual to the environment [4,41]. Our data also shows that IIV in behaviour is consistent at least to some degree between test-situations and that its genetic architecture is at least partly distinct from that of the main effects for those behaviours.

Intra-individual variation in the tonic immobility test did not correlate with the intra-individual variation observed in the other test situations. One possible explanation could be that individuals were adult when measured in the TI test and chicks when measured in the OF and SR test. Age has previously been shown to affect intra-individual behavioural variation in Red Junglefowls [58] perhaps because the cumulative experience of the environment leads to increasing consistency with age or because the neural circuitry has matured [53]. Yet, a large meta-analysis across animal species [3] found no difference in the repeatability of behaviour between juveniles and adults. Another explanation could be that the TI-test situation is different from the two other tests used in this study. In the TI-test, a test person physically restrains the bird to induce fear, whereas in the SR-test and OF-test, the test-person stays out of sight. Tonic immobility might therefore elicit a more direct fear response to a “predator”, whereas the SR-test and OF-test measures an animal’s anxiety levels when feeling exposed and alone. This would indicate that intra-individual variation in behaviour is trait specific, at least to some extent. It would therefore be interesting in future studies to explore intra-individual variation in behaviour across more direct fear inducing tests, to see if the magnitude of intra-individual behavioural variation in the TI-test is test or trait-specific.

In conclusion, this study demonstrates that intra-individual behavioural variation, like other behavioural traits, shows consistent differences between individuals and genotypes, even when animals are tested and reared in the same environment. This kind of behavioural variability between individuals has its own unique genetic architecture, separate from the behavioural traits it is based upon and therefore represents an important axis of consistent behavioural variation that evolution can act on. Our analysis also highlights six genes as putatively causative for intra-individual behavioural variation, four of which have been previously linked to behaviour and neural development.

## Figures and Tables

**Table 1:** Intra-individual behavioural variability correlations.

	OF_Move	OF_Velocity	OF_Centre_Freq	OF_Time_Centre	SR_Stimlatency	SR_Start	SR_Stim	SR_Distance	TI_Time	Global_Average	OF_Average	SR_Average	OF_SR_Average
**Trait—behavioral IIV**	magnitude	magnitude	magnitude	magnitude	magnitude	magnitude	magnitude	magnitude	magnitude	magnitude	magnitude	magnitude	magnitude
OF_move_magnitude	-	0.00 ***	0.00 ***	0.01 *	0.86	0.86	0.38	0.28	0.18	0.00 ***	0.00 ***	0.45	0.00 ***
OF_velocity_magnitude	0.87	-	0.00 ***	0.02 *	0.94	0.96	0.19	0.04 *	0.31	0.00 ***	0.00 ***	0.19	0.00 ***
OF_centre_freq_magnitude	0.42	0.45	-	0.00 ***	0.67	0.21	0.27	0.03 *	0.62	0.00 ***	0.00 ***	0.53	0.00 ***
OF_time_centre_magnitude	0.11	0.10	0.21	-	0.22	0.07#	0.07#	0.19	0.06#	0.00 ***	0.00 ***	0.02 *	0.00 ***
SR_stimlatency_magnitude	−0.01	0.00	−0.02	0.05	-	0.00 ***	0.00 ***	0.62	0.42	0.00 ***	0.81	0.00 ***	0.00 ***
SR_start_magnitude	0.01	0.00	−0.05	0.08	0.38	-	0.00 ***	0.70	0.55	0.00 ***	0.78	0.00 ***	0.00 ***
SR_stim_magnitude	0.04	0.06	0.05	0.08	0.64	0.24	-	0.57	1.00	0.00 ***	0.06	0.00 ***	0.00 ***
SR_distance_no_magnitude	0.05	0.09	0.10	0.06	−0.02	−0.02	0.02	-	0.32	0.00 ***	0.02 *	0.00 ***	0.00 ***
TI_time_magnitude	−0.06	−0.04	0.02	0.08	0.04	0.03	0.00	−0.04	-	0.00 ***	1.00	0.86	0.99
Global_average_magnitude	0.59	0.61	0.53	0.43	0.51	0.41	0.52	0.30	0.26	-	0.00 ***	0.00 ***	0.00 ***
OF_average_magnitude	0.83	0.84	0.72	0.49	0.01	0.01	0.08	0.10	0.00	0.75	-	0.06#	0.00 ***
SR_average_magnitude	0.03	0.06	0.03	0.10	0.79	0.63	0.75	0.39	0.01	0.68	0.08	-	0.00 ***
OF_SR_average_magnitude	0.62	0.64	0.53	0.42	0.51	0.41	0.54	0.32	0.00	0.97	0.77	0.70	-

Correlation between the magnitude of intra-individual behavioural variability, with *p*-values above the diagonal and correlation coefficients below the diagonal. Significant correlations are in bold and indicated with an asterisk (* indicate a *p*-value significant to *p* < 0.05, *** indicate a *p*-values significant to *p* < 0.001).

**Table 2:** QTL results of all IIV QTL as well as the standard trait QTL (based on individual trials and mean traits) from the same chromosome.

Trait	Chr	Position	Lod	R2	Add+/−s.e.	Dom+/−se	Lower ci	Upper ci	lower_Marker	Upper_Marker	Covariates	Interaction
OF_total_velocity_trial2	1	387	13.2	6.2	985+/−209	37.50+/−285	379	409	1_24012566	1_27115232	sex, batch, arena, w42,	1@387:10@82
OF_total_movement_trial2	1	428	11.3	6.5	−12.96+/−9.35	−15.60+/−11.01	401	453	Gg_rs14799859	1_30756049	sex, batch, arena, w42	10@64:1@428, 1@428:sex
OF_total_velocity_trial2	1	591	15.7	7.4	−2611+/−427	−2839+/−683	580	595	Gg_rs15239304	Gg_rs14815974	sex, batch, arena, w42,	1@591:13@32
SR_latency_to_enter_stimulus_zone_trial2	1	823	5.6	3.7	−128.57+/−28.38	−33.75+/−47.60	803	854	1_58811848	Gg_rs13874954	sex, batch, PC, w42	
SR_time_in_stimulus_zone_trial2	1	1088.6	4.9	3.2	−0.09+/−0.07	0.07+/−0.10	812	1117	1_58811848	1_84853091	arenas, sex, batch, PC, w42	
TI duration 2	1	1750	7.3	5	−30.8+/−11.7	−2.0+/−15.4	1735	1756	Gg_rs10728648	snp-23-342-18608-S-2	sex, batch, PC2,6	1@1748.0:7@3.0
**TI_duration_IIV**	**1**	**1750**	**12.1**	**10.1**	**−35.6+/−14.7**	**−28.9+/−9.5**	**1739**	**1755**	**Gg_rs10728648**	**snp-23-342-18608-S-2**	**batch, sex, PC1, PC2, PC3, PC10, w212**	**1@1750.0:17@159.0, 1@1750.0:3@564.0**
**OF_time_in_centre_zone_IIV**	**2**	**121**	**7.2**	**5.5**	**0.02+/−0.02**	**−0.03+/−0.03**	**96**	**137**	**Gg_rs15060526**	**Gg_rs15067636**	**batch, sex, w42, PC4, PC5, PC7**	**2@121.0:24@77.1**
TI duration 2	2	181	7.9	5.2	−63.0+/−14.9	−35.7+/−18.3	170	195	Gg_rs15070042	2_23979784	sex, batch, PC2,6	24@60.7:2@181.0
**OF_total_velocity_IIV**	**2**	**350**	**9.7**	**5.9**	**−78+/−51**	**−139+/−78**	**329**	**360**	**snp-5-242-106204-S-2**	**Gg_rs15094455**	**batch, sex, w42, PC**	**2@350.0:10@130.0**
**OF_total_movement_IIV**	**2**	**351**	**10.2**	**7.5**	**−0.38+/−0.14**	**−0.45+/−0.21**	**345**	**402**	**2_41000405**	**Gg_rs15094455**	**batch, sex, w42, PC1, PC10**	**2@351.0:10@130.0**
**OF_average_IIV**	**2**	**354**	**9.4**	**6.5**	**−0.14+/−0.04**	**−0.08+/−0.06**	**346**	**368**	**2_41000405**	**Gg_rs15094455**	**batch, sex, PC2, PC6, PC7**	**2@354.0:10@126.0**
OF_time_in_centre_zone_trial2	2	426	8.1	6.0	0.00+/−0.03	−0.08+/−0.03	416	431	Gg_rs15094455	GG_rs15099683	sex, batch, arena, w42,	2@426:4@191
OF_total_movement_trial2	2	513	4.1	2.3	0.81+/−0.21	−0.04+/−0.27	503	541	Gg_rs15107655	Gg_rs14200463	sex, batch, arena, w42	-
OF_total_movement_average	2	513	10.3	6.0	0.94+/−0.19	−0.56+/−0.24	504	520	Gg_rs15107655	Gg_rs15112090	sex, batch, w42	2@513:10@185
OF_total_movement_trial1	2	515	21	8.8	−3.54+/−0.91	2.85+/−1.24	504	522	Gg_rs15107655	Gg_rs15112090	sex, batch, w42	10@99:2@515, 2@515:w42
OF_number_visits_centre_trial2	2	519	5.42	3.57	3.62+/−0.73	−1.27+/−1.07	505	568	Gg_rs15107655	Gg_rs14206130	sex, batch, arena, w42,	-
SR_time_in_start_zone_average	2	656	13.2	6.7	−0.07+/−0.02	0.03+/−0.02	647	662	Gg_rs10723221	Gg_rs14225365	arenas, sex, batch, PC, w42	18@54.0:2@656.0, 2@656.0:6@138.0
SR_latency_to_enter_stimulus_zone_trial2	2	657.6	3.8	2.5	−26.05+/−6.96	24.95+/−9.58	512	715	Gg_rs15107655	Gg_rs14232962	sex, batch, PC, w42	
SR_latency_to_enter_stimulus_zone_average	2	658	5.4	3.4	−26.32+/−5.53	18.97+/−7.61	652	664	Gg_rs10723221	Gg_rs14225365	arenas, sex, batch, PC, w42	
TI average d.	2	774	6.5	4.6	−10.8+/−9.2	−28.7+/−14	764	795	RBL1120	Gg_rs15146557	sex, batch, PC2,6	2@774.0:24@60.7
SR_latency_to_enter_stimulus_zone_average	3	162	4.9	3.1	7.86+/−5.41	39.48+/−8.52	137	180	Gg_rs14084016	Gg_rs15282380	arenas, sex, batch, PC, w42	
SR_latency_to_enter_stimulus_zone_trial2	3	166	4.6	3.0	3.21+/−6.83	48.13+/−10.65	141	179	Gg_rs14084016	3_16300000	sex, batch, PC, w42	
**SR_time_in_stimulus_zone_IIV**	**3**	**408**	**9.6**	**7.0**	**3.5+/−2.3**	**−7.4+/−2.8**	**401**	**411**	**Gg_rs15337358**	**Gg_rs14349767**	**batch, sex, w42, PC10**	**3@407.9:20@112.0**
**OF_total_velocity_IIV**	**3**	**417**	**6.3**	**3.8**	**352+/−86**	**56+/−112**	**413**	**432**	**Gg_rs14349767**	**Gg_rs15361114**	**batch, sex, w42, PC**	**3@417.0:7@104.0**
**OF_total_movement_IIV**	**3**	**419**	**5.8**	**4.2**	**2.0+/−0.66**	**0.05+/−0.80**	**411**	**431**	**Gg_rs14349767**	**snp-17-141-24973-S-1**	**batch, sex, w42, PC1, PC10**	**3@419.0:sex**
**TI_duration_IIV**	**3**	**564**	**5.6**	**4.5**	**−8.8+/−15.6**	**−8.3+/−19.3**	**443**	**578**	**rbl1045**	**Gg_rs15416272**	**batch, sex, PC1, PC2, PC3, PC10, w212**	**1@1750.0:3@564.0**
TI duration 2	7	4	11.5	7.8	11.2+/−13.5	48.5+/−17.7	0	8	Gg_rs15826188	Gg_rs15828492	sex, batch, PC2,6	1@1748.0:7@3.0, 7@3.0:24@60.7
OF_total_movement_average	7	103	5.0	2.8	0.62+/−0.25	−0.54+/−0.31	92	110	Gg_rs15835348	Gg_rs15845344	sex, batch, w42	10@99.2:7@103
OF_total_velocity_average	7	104	6.8	3.1	387.30+/−179.98	−322.00+/−228	95	110	Gg_rs15835348	Gg_rs15845344	sex, batch, w42	7@104:10@101
**OF_total_velocity_IIV**	**7**	**104**	**7.1**	**7.1**	**462+/−91**	**−498+/−124**	**86**	**113**	**Gg_rs15834580**	**Gg_rs15845344**	**batch, sex, w42,**	**3@417.0:7@104.0**
**global_IIV**	**7**	**122**	**5.2**	**3.8**	**−0.37+/−0.31**	**0.81+/−0.35**	**113**	**128**	**Gg_rs14607135**	**GG_rs15846462**	**batch, sex, PC1, PC4, PC7, PC8**	**7@122.0:sex**
**OF_average_IIV**	**7**	**122**	**5.8**	**4**	**0.03+/−0.46**	**0.70+/−0.53**	**114**	**128**	**Gg_rs14607135**	**GG_rs15846462**	**batch, sex, PC2, PC6, PC7**	**7@122.0:sex**
OF_number_visits_centre_trial1	7	145	4.9	3.5	−15.26+/−11.13	33.33+/−12.48	137	153	7_15017822	Gg_rs15853763	sex, batch, arena, w42,	-
SR_latency_to_enter_stimulus_zone_average	7	175.5	8.5	5.5	−15.27+/−7.39	1.91+/−9.12	159	194	Gg_rs15850767	7_24150587	arenas, sex, batch, PC, w42	7@175.5:23@124.0
SR_time_in_stimulus_zone_trial2	7	281	5.6	3.6	0.11+/−0.06	−0.32+/−0.09	272	295	Gg_rs15878354	Gg_rs13600357	arenas, sex, batch, PC, w42	
OF_total_movement_trial2	10	64	15.1	8.9	13.43+/−8.52	−11.56+/−8.74	46	177	Gg_rs14941298	Gg_rs14949856	sex, batch, arena, w42	10@64:1@428, 10@64:sex
OF_total_velocity_trial2	10	82	11.2	5.2	−47+/−228	−42.70+/−347	67	111	Gg_rs14941656	Gg_rs14003134	sex, batch, arena, w42,	1@387:10@82
OF_total_movement_trial1	10	99	14.2	13.7	1.20+/−0.81	2.35+/−1.18	94	104	Gg_rs14001676	Gg_rs14003134	sex, batch, w42	10@99:2@515, 10@99:w42
TI average d.	10	99	5.9	4.1	−15.1+/−7.7	14.9+/−10.1	86	109	Gg_rs14941656	Gg_rs14003134	sex, batch, PC2,6	10@99.0:20@247.7
OF_total_movement_average	10	99	7.2	4.1	0.73+/−0.19	−0.76+/−0.30	98	106	Gg_rs14001865	Gg_rs14003134	sex, batch, w42	10@99.2:7@103
OF_total_velocity_trial1	10	99	4.7	3.0	528.18+/−134.16	−619.00+/−192	95	110	Gg_rs15060526	Gg_rs14139143	sex, batch, w42	-
OF_total_velocity_average	10	101	9.9	4.6	649+/−150	−1006.00+/−230	96	107	Gg_rs14001676	Gg_rs14003134	sex, batch, w43	7@104:10@101
**OF_average_IIV**	**10**	**126**	**8.5**	**5.8**	**0.05+/−0.05**	**−0.15+/−0.07**	**107**	**134**	**Gg_rs14002026**	**Gg_rs14006050**	**batch, sex, PC2, PC6, PC7**	**2@354.0:10@126.0**
**OF_total_movement_IIV**	**10**	**130**	**8.9**	**6.5**	**0.16+/−0.15**	**−0.38+/−0.22**	**118**	**139**	**Gg_rs14003134**	**Gg_rs14006050**	**batch, sex, w42, PC1, PC10**	**2@351.0:10@130.0**
**OF_total_velocity_IIV**	**10**	**130**	**9.9**	**9.9**	**84+/−52**	**−175+/−82**	**123**	**138**	**10_9525779**	**Gg_rs14006050**	**batch, sex, w42,**	**2@350.0:10@130.0**
OF_total_movement_average	10	185	8.2	4.7	0.72+/−0.19	−0.64+/−0.24	178	192	Gg_rs14008254	GG_rs14951592	sex, batch, w42	2@513:10@185
TI duration 1	10	185	8.5	6.8	2.4+/−9.5	−34.6+/−12.2	176	198	Gg_rs14008254	GG_rs14951592	sex, batch, PC1,4	6@258.7:10@185.0
OF_total_velocity_average	17	1	15.0	7.2	611+/−129	−473.00+/−174	0	8	Gg_rs15035175	Gg_rs15033588	sex, batch, w44	17@1:8@194
**TI_duration_IIV**	**17**	**159**	**7.4**	**6**	**28.9+/−9.5**	**42.0+/−13.3**	**132**	**178**	**Gg_rs14099239**	**Gg_rs14097200**	**batch, sex, PC1, PC2, PC3, PC10, w212**	**1@1750.0:17@159.0**
SR_time_in_start_zone_average	18	54	6.4	3.2	0.02+/−0.02	−0.05+/−0.02	24	63	18_3045583	Gg_rs13507726	arenas, sex, batch, PC, w42	18@54.0:2@656.0
**SR_time_in_stimulus_zone_IIV**	**20**	**112**	**10.3**	**7.6**	**6.9+/−3.1**	**−1.1+/−2.9**	**60**	**145**	**GG_rs15171843**	**Gg_rs15175145**	**batch, sex, w42, PC10**	**3@407.9:20@112.0**
SR_time_in_stimulus_zone_trial2	20	245	6.5	4.2	0.01+/−0.02	−0.01+/−0.03	239	252	Gg_rs15177950	Gg_rs14280872	arenas, sex, batch, PC, w42	
TI average d.	20	247.7	6.4	4.8	−9.4+/−7.1	24+/−10.1	237	252	Gg_rs15177950	Gg_rs14280872	sex, batch, PC2,6	10@99.0:20@247.7
TI average d.	24	60.7	7	4.9	−20.9+/−8.5	−21.0+/−11	53	67	GG_rs16194400	Gg_rs14294768	sex, batch, PC2,6	2@774.0:24@60.7
TI duration 2	24	61	16.5	11.5	21.5+/−15.3	−40.9+/−18.7	54	65	GG_rs16194400	Gg_rs14294768	sex, batch, PC2,6	7@3.0:24@60.7, 24@60.7:2@181.0
SR_total_velocity_trial1	24	72	5.2	2.3	370+/−99	−607+/−127	66	77	Gg_rs13604720	Gg_rs14295223	arenas, sex, batch, PC, w42	
SR_total_movement_trial1	24	75	5.6	3.7	0.54+/−0.13	−0.80+/−0.17	66	80	Gg_rs13604720	Gg_rs15219713	arenas, sex, batch, PC, w42	
**OF_time_in_centre_zone_IIV**	**24**	**77**	**8.8**	**6.8**	**−0.01+/−0.02**	**0.09+/−0.02**	**73**	**79**	**Gg_rs14294768**	**Gg_rs15219713**	**batch, sex, w42, PC4, PC5, PC7**	**2@121.0:24@77.1**

Location (in cm), LOD score, additive and dominance effects, 95% confidence interval in cm, covariates used and r-squared (effect size %) are all included. IIV QTL are highlighted in blue (for QTL that do not overlap with any main effects QTL) and in grey (for QTL that do overlap main effects QTL).

**Table 3:** Candidate genes and causality scores.

QTL Chromosome and Position (cM)	Gene	SINGLE MODEL		COMBINED MODEL		NEO	
		Gene Expression (*p*-Value)	Genotype (*p*-Value)	Gene Expression (*p*-Value)	Genotype (*p*-Value)	LEO Score	Model *p*-Value
chr 3—419	MAP7	0.03/0.06	0.02	0.04	0.03	0.36	0.09
chr 2—351	SFRP4	0.02/0.05	NS	NA	NA	0.29	0.92
chr 3—417	MAP7	0.02/0.04	0.12	0.02	0.11	0.23	0.06
chr 2—350	SFRP4	0.01/0.03	0.05	0.02	0.05	0.5	0.84
chr 10—130	X603600179F1 (LOC100136711)	0.04/0.04	NS	NA	NA	0.51	0.72
chr 2—35	X603862030F1(TjP1)	0.07/0.07	NS	NA	NA	−0.33	0.36
chr 2—354	SFRP4	0.02/0.07	NS	NA	NA	0.16	0.07
chr 3—408	X603599288F1(unknown)	0.009/0.02	0.06/0.03	0.016	0.05/0.02	0.53	0.57
chr 3—408	X603600684F1(GALNT2)	0.03/0.06	0.06/0.03	0.02	0.05/0.02	0.27	0.31
chr 1—1750	ITGBL1	0.005/0.005	0.03	0.005	0.14	0.51	0.33
chr 1—1750	ABHD13	0.05/0.05	0.03	0.04	0.09	−0.11	0.17
chr 3—564	ENPP1	0.02/0.04	NS	NA	NA	0.34	0.26
chr 7—122	X603601250F1(C2orf42)	0.04/0.07	0.04	0.03	0.1	0.25	<0.001

The intra-individual behavioural variability (IIV) QTL location (chromosome) and position (in cm) is provided. *p*-values are given for the significance of the gene expression and genotype (QTL) models, as predicting IIV. The single model *p*-values show these effects individually, while the combined model fits both gene expression and genotype simultaneously. For gene expression (single model), the nominal *p*-value and the *p*-value after multiple testing correction are both given. *p*-values > 0.2 are given as N.S. (non-significant). The NEO causality score (LEO score) and the *p*-values of the causal model are also provided for each gene.

## Supplementary Materials

Supplementary materials can be found at https://www.mdpi.com/1422-0067/21/21/8069/s1.
