# Supplementary material for: Intra-Individual Behavioural Variability: A Trait under Genetic Control
Source: Int J Mol Sci. 2020 Oct 29;21(21):8069. doi: 10.3390/ijms21218069 (PMC7663371; doi:10.3390/ijms21218069)
Supplement: Supplementary file 1 [file ijms-21-08069-s001.pdf]

SUPPLEMENTARY MATERIAL

Behavioural predictability: a trait under genetic control

Supplementary Table 1. IIIV trait means and standard deviations

IIV trait	OF_number_visits_centre_IIV	OF_time_in_centre_zone_IIV	OF_total_movement_IIV	OF_total_velocity_IIV	SR_latency_to_enter_stimulus_zone_IIV	SR_time_in_start_zone_IIV	SR_time_in_stimulus_zone_IIV	SR_total_movement_IIV	TI_duration_IIV	OF_average_IIV	SR_average_IIV	OF_SR_average_IIV	Global_average_IIV
**mean**	8.60	2.40E-01	2.46	8.24E+02	9.60E+01	2.46E-01	2.91E-01	1.49	1.22E+02	-2.68E-03	1.58E-03	2.46E-03	-4.22E-03
**S.D.**	8.28	2.11E-01	2.07	8.32E+02	8.95E+01	2.65E-01	2.54E-01	1.32	1.32E+02	7.21E-01	6.10E-01	4.92E-01	4.60E-01

Supplementary Table 2. Table of IIV QTL and all standard trait trial and mean QTL (all chromosomes) for comparison.

trait	chr	position	LOD	add+/-s.e.	dom+/-se	lower CI	upper CI	lower_marker	upper_marker	covariates	interaction	R2
OF_total_velocity_trial2	1	387	13.2	985+/-209	37.50+/-285	379	409	1_24012566	1_27115232	sex, batch, arena, w42,	1@387:10@82	6.2
OF_total_movement_trial2	1	428	11.3	-12.96+/-9.35	-15.60+/-11.01	401	453	Gg_rs14799859	1_30756049	sex,batch,arena,w42	10@64:1@428, 1@428:sex	6.5
OF_total_velocity_trial2	1	591	15.7	-2611+/-427	-2839+/-683	580	595	Gg_rs15239304	Gg_rs14815974	sex, batch, arena, w42,	1@591:13@32	7.4
SR_latency_to_enter_stimulus_zone_trial2	1	823	5.3	-127.61+/-28.63	-33.54+/-47.69	803	855	1_58811848	Gg_rs13874954	sex,batch,arena		3.5
SR_time_in_stimulus_zone_trial2	1	1088.6	5.7	-0.08+/-0.07	0.04+/-0.10	1082	1111	snp-248-19-898-S-2	1_84853091	sex,batch,arena		3.7
TI duration 2	1	1750	7.3	-30.8+/-11.7	-2.0+/-15.4	1735	1756	Gg_rs10728648	snp-23-342-18608-S-2	sex, batch, PC2,6	1@1748.0:7@3.0	5
TI_duration_IIV	1	1750	12.1	-35.6+/-14.7	-28.9+/-9.5	1739	1755	Gg_rs10728648	snp-23-342-18608-S-2	batch, sex, PC1, PC2, PC3, PC10, w212	1@1750.0:17@159.0, 1@1750.0:3@564.0	10.1
OF_time_in_centre_zone_IIV	2	121	7.2	0.02+/-0.02	-0.03+/-0-03	96	137	Gg_rs15060526	Gg_rs15067636	batch,sex,w42, PC4, PC5, PC7	2@121.0:24@77.1	5.5
TI duration 2	2	181	7.9	-63.0+/-14.9	-35.7+/-18.3	170	195	Gg_rs15070042	2_23979784	sex, batch, PC2,6	24@60.7:2@181.0	5.2
OF_total_velocity_IIV	2	350	9.7	-78+/-51	-139+/-78	329	360	snp-5-242-106204-S-2	Gg_rs15094455	batch,sex,w42, PC	2@350.0:10@130.0	5.9
OF_total_movement_IIV	2	351	10.2	-0.38+/-0.14	-0.45+/-0.21	345	402	2_41000405	Gg_rs15094455	batch, sex, w42, PC1,PC10	2@351.0:10@130.0	7.5
OF_average_IIV	2	354	9.4	-0.14+/-0.04	-0.08+/-0.06	346	368	2_41000405	Gg_rs15094455	batch, sex, PC2, PC6, PC7	2@354.0:10@126.0	6.5
OF_time_in_centre_zone_trial2	2	426	8.1	0.00+/-0.03	-0.08+/-0.03	416	431	Gg_rs15094455	GG_rs15099683	sex, batch, arena, w42,	2@426:4@191	6.0
OF_total_movement_trial2	2	513	4.1	0.81+/-0.21	-0.04+/-0.27	503	541	Gg_rs15107655	Gg_rs14200463	sex,batch,arena,w42	-	2.3
OF_total_movement_average	2	513	10.3	0.94+/-0.19	-0.56+/-0.24	504	520	Gg_rs15107655	Gg_rs15112090	sex, batch, w42	2@513:10@185	6.0
OF_total_movement_trial1	2	515	21	-3.54+/-0.91	2.85+/-1.24	504	522	Gg_rs15107655	Gg_rs15112090	sex,batch,w42	10@99:2@515, 2@515:w42	8.8
OF_number_visits_centre_trial2	2	519	5.42	3.62+/-0.73	-1.27+/-1.07	505	568	Gg_rs15107655	Gg_rs14206130	sex, batch, arena, w42,	-	3.57
SR_latency_to_enter_stimulus_zone_trial2	2	657	5.8	-31.60+/-7.26	24.53+/-9.75	651	664	Gg_rs10723221	Gg_rs14225365	sex,batch,arena	2@657.6:3@174.0	3.8
SR_time_in_start_zone_average	2	657.6	13.6	-0.07+/-0.02	0.04+/-0.02	652	661	Gg_rs10723221	Gg_rs14225365	sex,batch,w42	18@55.0:2@657.6,2@657.6:6@146.0	7.1
SR_latency_to_enter_stimulus_zone_average	2	658	5.6	-27.21+/-5.51	18.87+/-7.60	652	665	Gg_rs10723221	Gg_rs14225365	sex,batch,w42		3.7
TI average d.	2	774	6.5	-10.8+/-9.2	-28.7+/-14	764	795	RBL1120	Gg_rs15146557	sex, batch, PC2,6	2@774.0:24@60.7	4.6
SR_latency_to_enter_stimulus_zone_average	3	143	5	6.05+/-5.34	39.89+/-8.46	136	180	Gg_rs14084016	3_16300000	sex,batch,w42		3.2
SR_latency_to_enter_stimulus_zone_trial2	3	174	6.4	5.46+/-7.19	42.20+/-10.18	142	179	Gg_rs14084016	3_16300000	sex,batch,arena	2@657.6:3@174.0	4.2
SR_time_in_stimulus_zone_IIV	3	408	9.6	3.5+/-2.3	-7.4+/-2.8	401	411	Gg_rs15337358	Gg_rs14349767	batch,sex,w42,PC10	3@407.9:20@112.0	7.0
OF_total_velocity_IIV	3	417	6.3	352+/-86	56+/-112	413	432	Gg_rs14349767	Gg_rs15361114	batch,sex,w42, PC	3@417.0:7@104.0	3.8
OF_total_movement_IIV	3	419	5.8	2.0+/-0.66	0.05+/-0.80	411	431	Gg_rs14349767	snp-17-141-24973-S-1	batch, sex, w42, PC1,PC10	3@419.0:sex	4.2
TI_duration_IIV	3	564	5.6	-8.8+/-15.6	-8.3+/-19.3	443	578	rbl1045	Gg_rs15416272	batch, sex, PC1, PC2, PC3, PC10, w212	1@1750.0:3@564.0	4.5
OF_time_in_centre_zone_trial2	4	191	5.4	0.01+/-0.02	0.04+/-0.03	89	201	Gg_rs15481407	Gg_rs15522441		2@426:4@191	3.9
TI duration 1	4	283	9.7	33.4+/-10.5	11.4+/-16.2	272	292	Gg_rs14446625	4_37860292		4@283.0:15@187.9	7.8
OF_total_movement_trial2	4	360	7.1	-1.12+/-0.51	1.53+/-0.90	343	378	Gg_rs14457643	Gg_rs14470123		10@64:4@360	4
SR_time_in_start_zone_trial1	4	434	5	0.08+/-0.03	-0.04+/-0.03	428	445	Gg_rs15603543	Gg_rs14487406			3.5
SR_time_in_stimulus_zone_trial1	4	437	3.5	-0.06+/-0.03	0.05+/-0.03	427	458	Gg_rs15603543	chr4:71833345			*2.4*
SR_latency_to_enter_stimulus_zone_trial1	4	438	4.8	31.24+/-9.13	-11.12+/-12.36	428	458	Gg_rs15603543	chr4:71833345			3.5
SR_time_in_start_zone_average	5	95.6	9.1	0.05+/-0.01	-0.05+/-0.02	79	108	5_5571125	Gg_rs14518923		12@53.0:5@95.6	4.7
OF_number_visits_centre_trial2	6	69	5.65	7.65+/-2.01	-3.66+/-2.73	41	77	6_2930562	Gg_rs15765462		6@69:sex	3.72
SR_time_in_start_zone_average	6	146	13.3	-0.04+/-0.02	0.01+/-0.04	124	175	Gg_rs15773041	Gg_rs15781634		2@657.6:6@146.0, 6@146.0:16@0.0	6.9
TI duration 1	6	259	6.9	5.6+/-9.1	10.7+/-12.2	247	270	6_25762392	Gg_rs14592224		6@258.7:10@185.0	5.5
TI duration 2	7	4	11.5	11.2+/-13.5	48.5+/-17.7	0	8	Gg_rs15826188	Gg_rs15828492	sex, batch, PC2,6	1@1748.0:7@3.0, 7@3.0:24@60.7	7.8
OF_total_movement_average	7	103	5.0	0.62+/-0.25	-0.54+/-0.31	92	110	Gg_rs15835348	Gg_rs15845344	sex, batch, w42	10@99.2:7@103	2.8
OF_total_velocity_average	7	104	6.8	387.30+/-179.98	-322.00+/-228	95	110	Gg_rs15835348	Gg_rs15845344	sex, batch, w42	7@104:10@101	3.1
OF_total_velocity_IIV	7	104	7.1	462+/-91	-498+/-124	86	113	Gg_rs15834580	Gg_rs15845344	batch,sex,w42,	3@417.0:7@104.0	7.1
global_IIV	7	122	5.2	-0.37+/-0.31	0.81+/-0.35	113	128	Gg_rs14607135	GG_rs15846462	batch, sex, PC1, PC4,PC7,PC8	7@122.0:sex	3.8
OF_average_IIV	7	122	5.8	0.03+/-0.46	0.70+/-0.53	114	128	Gg_rs14607135	GG_rs15846462	batch, sex, PC2, PC6, PC7	7@122.0:sex	4
OF_number_visits_centre_trial1	7	145	4.9	-15.26+/-11.13	33.33+/-12.48	137	153	7_15017822	Gg_rs15853763	sex, batch, arena, w42,	-	3.5
SR_latency_to_enter_stimulus_zone_average	7	176	8.2	-15.54+/-7.38	1.52+/-9.15	158	193	Gg_rs15850767	7_24150587	sex,batch,w42	7@176.0a:23@122.0	5.4
SR_time_in_stimulus_zone_trial2	7	281	6	0.11+/-0.06	-0.32+/-0.09	271	293	Gg_rs15878354	Gg_rs13600357	sex,batch,arena		3.9
OF_total_velocity_average	8	194	13.6	744+/-167	-1070.00+/-297	183	207	Gg_rs14652282	Gg_rs15935538	sex, batch, w45	17@1:8@194	6.5
OF_total_movement_trial2	10	64	15.1	13.43+/-8.52	-11.56+/-8.74	46	177	Gg_rs14941298	Gg_rs14949856	sex,batch,arena,w42	10@64:1@428, 10@64:sex	8.9
OF_total_velocity_trial2	10	82	11.2	-47+/-228	-42.70+/-347	67	111	Gg_rs14941656	Gg_rs14003134	sex, batch, arena, w42,	1@387:10@82	5.2
TI average d.	10	99	5.9	-15.1+/-7.7	14.9+/-10.1	86	109	Gg_rs14941656	Gg_rs14003134	sex, batch, PC2,6	10@99.0:20@247.7	4.1
OF_total_movement_trial1	10	99	14.2	1.20+/-0.81	2.35+/-1.18	94	104	Gg_rs14001676	Gg_rs14003134	sex,batch,w42	10@99:2@515, 10@99:w42	13.7
OF_total_velocity_trial1	10	99	4.7	528.18+/-134.16	-619.00+/-192	95	110	Gg_rs15060526	Gg_rs14139143	sex, batch, w42	-	3.0

OF_total_movement_average	10	99	7.2	0.73+/-0.19	-0.76+/-0.30	98	106	Gg_rs14001865	Gg_rs14003134	sex, batch, w42	10@99.2:7@103	4.1
OF_total_velocity_average	10	101	9.9	649+/-150	-1006.00+/-230	96	107	Gg_rs14001676	Gg_rs14003134	sex, batch, w43	7@104:10@101	4.6
**OF_average_IIV**	**10**	**126**	**8.5**	**0.05+/-0.05**	**-0.15+/-0.07**	**107**	**134**	**Gg_rs14002026**	**Gg_rs14006050**	**batch, sex, PC2, PC6, PC7**	**2@354.0:10@126.0**	**5.8**
**OF_total_movement_IIV**	**10**	**130**	**8.9**	**0.16+/-0.15**	**-0.38+/-0.22**	**118**	**139**	**Gg_rs14003134**	**Gg_rs14006050**	**batch, sex, w42, PC1,PC10**	**2@351.0:10@130.0**	**6.5**
**OF_total_velocity_IIV**	**10**	**130**	**9.9**	**84+/-52**	**-175+/-82**	**123**	**138**	**10_9525779**	**Gg_rs14006050**	**batch,sex,w42,**	**2@350.0:10@130.0**	**9.9**
OF_total_movement_average	10	185	8.2	0.72+/-0.19	-0.64+/-0.24	178	192	Gg_rs14008254	GG_rs14951592	sex, batch, w42	2@513:10@185	4.7
TI duration 1	10	185	8.5	2.4+/-9.5	-34.6+/-12.2	176	198	Gg_rs14008254	GG_rs14951592	sex, batch, PC1,4	6@258.7:10@185.0	6.8
SR_time_in_stimulus_zone_trial1	11	3	9.1	0.02+/-0.02	-0.13+/-0.03	0	11	Gg_rs14957923	Gg_rs14019836	sex,batch,arena	12@175.0:11@3.0	6.3
SR_time_in_stimulus_zone_trial2	12	3.4	7.1	-0.04+/-0.02	-0.06+/-0.03	0	11	Gg_rs14031213	Gg_rs14971198	sex,batch,arena	20@246.0:12@3.4	4.6
SR_time_in_start_zone_average	12	53	8.2	-0.02+/-0.02	0.03+/-0.02	44	69	Gg_rs13621493	Gg_rs14974529	sex,batch,w42	12@53.0:5@95.6	4.2
SR_time_in_stimulus_zone_trial1	12	175	7.5	0.00+/-0.02	0.13+/-0.04	160	185	Gg_rs14040823	12_14051161	sex,batch,arena	12@175.0:11@3.0	5.1
OF_total_velocity_trial2	13	32	15.0	38.92+/-684.03	557.00+/-1083	28	40	GG_rs15676474	Gg_rs14991095	sex, batch, arena, w42,	1@591:13@32	7.1
TI duration 1	15	188	7.9	-18.0+/-9.6	-19.5+/-12.3	177	189	Gg_rs14095161	Gg_rs14095923	sex, batch, PC1,4	4@283.0:15@187.9	6.3
SR_time_in_start_zone_average	*16*	*0*	*6.9*	*0.03+/-0.01*	*-0.02+/-0.02*	0	1	Gg_rs15026773	Gg_rs14096699	sex,batch,w42	*6@146.0:16@0.0*	3.5
OF_total_velocity_average	17	1	15.0	611+/-129	-473.00+/-174	0	8	Gg_rs15035175	Gg_rs15033588	sex, batch, w44	17@1:8@194	7.2
**TI_duration_IIV**	**17**	**159**	**7.4**	**28.9+/-9.5**	**42.0+/-13.3**	**132**	**178**	**Gg_rs14099239**	**Gg_rs14097200**	**batch, sex, PC1, PC2, PC3, PC10, w212**	**1@1750.0:17@159.0**	**6**
SR_time_in_start_zone_average	18	55	7	0.03+/-0.02	-0.06+/-0.02	33	64	18_3045583	Gg_rs13507726	sex,batch,w42	18@55.0:2@657.6	3.5
**SR_time_in_stimulus_zone_IIV**	**20**	**112**	**10.3**	**6.9+/-3.1**	**-1.1+/-2.9**	**60**	**145**	**GG_rs15171843**	**Gg_rs15175145**	**batch,sex,w42,PC10**	**3@407.9:20@112.0**	**7.6**
SR_time_in_stimulus_zone_trial2	20	245	6.4	0.01+/-0.02	0.00+/-0.03	239	252	Gg_rs15177950	Gg_rs14280872	sex,batch,arena	20@246.0:12@3.4	4.2
TI average d.	20	247.7	6.4	-9.4+/-7.1	24+/-10.1	237	252	Gg_rs15177950	Gg_rs14280872	sex, batch, PC2,6	10@99.0:20@247.7	4.8
SR_latency_to_enter_stimulus_zone_average	23	122	7.1	-10.36+/-6.55	7.76+/-9.61	108	136	RBL4501	23_4455543	sex,batch,w42	**7@176.0a:23@122.0**	4.7
TI average d.	24	60.7	7	-20.9+/-8.5	-21.0+/-11	53	67	GG_rs16194400	Gg_rs14294768	sex, batch, PC2,6	2@774.0:24@60.7	4.9
TI duration 2	24	61	16.5	21.5+/-15.3	-40.9+/-18.7	54	65	GG_rs16194400	Gg_rs14294768	sex, batch, PC2,6	7@3.0:24@60.7, 24@60.7:2@181.0	11.5
SR_total_velocity_trial1	24	73	5.4	364.03+/-98.99	-619.41+/-126.47	65	80	Gg_rs13604720	Gg_rs15219713	sex,batch,arena, w42		2.5
SR_total_movement_trial1	24	75	6.1	0.57+/-0.13	-0.85+/-0.17	66	84	Gg_rs13604720	Gg_rs13605511	sex,batch,arena, w42		4.1
**OF_time_in_centre_zone_IIV**	**24**	**77**	**8.8**	**-0.01+/-0.02**	**0.09+/-0.02**	**73**	**79**	**Gg_rs14294768**	**Gg_rs15219713**	**batch,sex,w42, PC4, PC5, PC7**	**2@121.0:24@77.1**	**6.8**

Supplementary Table 3. IIV QTL with mean trait included as a covariate

Trait - behavioral IIV	QTL chromosome and position (cM)	LOD	r-sq	additive effect	additive se	dominance effect	dominance se	lower C.I.	upper C.I.	interactions
OF_total_movement_IIV	chr 3 - 419	4.8	2.8	1.74	0.61	0.09	0.74	385	430	sex
OF_total_movement_IIV	chr 2 - 353	9.2	5.5	-0.27	0.13	-0.33	0.18	338	368	chr2xchr10
OF_total_movement_IIV	chr 10 - 130	8.9	5.3	0.11	0.14	-0.42	0.2	123	138	chr2xchr10
OF_total_movement_IIV	chr 7 - 121	5.9	3.5	-0.99	1.23	3.21	1.45	114	126	sex
OF_time_in_centre_zone_IIV	chr 2 - 121	6.7	5.5	0.02	0.02	-0.03	0.03	95	138	chr2 x chr24
OF_time_in_centre_zone_IIV	chr 24 - 77.1	8.8	6.8	-0.01	0.02	0.09	0.02	73	79	chr2 x chr24
OF_total_velocity_IIV	chr 2 - 350	9.9	4.7	-37	45	-116	70	337	364	chr2xchr10
OF_total_velocity_IIV	chr 10 - 132	10.4	5	39	50	-175	71	124	144	chr2xchr10
OF_total_velocity_IIV	ch 3 - 417	6.5	3	303	74	23	101	402	442	chr3 x chr7
OF_total_velocity_IIV	chr 7 - 109	5.4	2.6	386	84	-329	110	105	118	chr3 x chr7
SR_time_in_stimulus_zone_IIV	chr 3 - 408	9.1	6.4	0.03	0.02	-0.08	0.03	403	411	chr3 x chr20
SR_time_in_stimulus_zone_IIV	chr 20 - 112	9.6	6.9	0.06	0.02	-0.11	0.03	108	126	chr3 x chr20
TI_duration_IIV	chr 1 - 1734	6.1	3.5	-17.9	20.6	49.9	31.7	1720	1748	chr1 x chr3
TI_duration_IIV	chr 3 - 451	6.5	3.8	-40.1	16.6	43.8	18.4	447	453	chr1 x chr3
